# Pharmacokinetic/Pharmacodynamic Modeling of Tulathromycin against *Pasteurella multocida* in a Porcine Tissue Cage Model

**DOI:** 10.3389/fphar.2017.00392

**Published:** 2017-06-28

**Authors:** Qiaoyi Zhou, Guijun Zhang, Qin Wang, Wenguang Liu, Yan Huang, Pengling Yu, Yanqin Li, Huanzhong Ding, Binghu Fang

**Affiliations:** ^1^National Reference Laboratory of Veterinary Drug Residues, College of Veterinary Medicine, South China Agricultural UniversityGuangzhou, China; ^2^Guangdong Wens Dahuanong Biotechnology Co., Ltd.Yunfu, China

**Keywords:** tulathromycin, *Pasteurella multocida*, *ex vivo* PK/PD, tissue cage model, PK/PD parameters

## Abstract

Tulathromycin, a macrolide antibiotic, is used for the treatment of respiratory disease in cattle and swine. The aim of our study was to investigate the *in vitro* and *ex vivo* activities of tulathromycin in serum, (non-inflamed) transudate, and (inflamed) exudate against *Pasteurella multocida* in piglets. The pharmacokinetics properties of tulathromycin were studied for serum, transudate, and exudate using a tissue cage model. *In vitro* antibiotic susceptibility of *P. multocida* and dynamic time-kill curve experiments over eight tulathromycin concentrations were determined. The ratio of 24-h area under the concentration–time curve to minimum inhibitory concentration [AUC_(0-24 h)_/MIC] was recognized as an important pharmacokinetic/pharmacodynamic (PK/PD) parameter of tulathromycin for antibacterial efficiency (*R*^2^ = 0.9969). In serum *ex vivo*, for bacteriostatic, bactericidal activity, and virtual bacterial eradication AUC_(0-24 h)_/MIC values for tulathromycin were 44.55, 73.19, and 92.44 h by using sigmoid *E*_max_ model WinNonlin software, respectively, and lower values were obtained for exudate and transudate. In conjunction with the data on MIC_90_, the dose of tulathromycin for a bacteriostatic effect and virtual elimination of *P. multocida* as computed using the value of the PK/PD breakpoint obtained in serum were 6.39 and 13.25 mg/kg. However, it would be preferable to calculate a dose combined with population pharmacokinetics data to optimize the dosage regimen for bacteriological and clinical cure.

## Introduction

Tulathromycin (Draxxin^®^), a semi-synthetic macrolide developed by Pfizer, is approved for the treatment of bacterial infection causing bovine respiratory disease (BRD) and swine respiratory disease (SRD) (Evans, 2006). Multiple laboratory studies have shown that tulathromycin exhibits high efficacy against common bacterial pathogens causative of respiratory disease in cattle and swine (Godinho et al., 2005; Hart et al., 2006), and the drug is associated with rapid absorption, high bioavailability, a long elimination half-life, significant tissue distribution, and high cell infiltration (Benchaoui et al., 2004; Nowakowski et al., 2004). Studies *in vitro* with *Mannheimia haemolytica* (*M. haemolytica*) and *Pasteurella multocida* (*P. multocida*) indicate that tulathromycin has a concentration–effect relationship in Mueller-Hinton broth (MHB) and serum (Toutain et al., 2017). It has been proposed that the best predictor of efficacy for tulathromycin is the ratio of area under the concentration–time curve to minimum inhibitory concentration (AUC/MIC) (Evans, 2005).

Many scholars believe that pharmacokinetic (PK)/pharmacodynamic (PD) model is an important tool to improve the efficacy of antibacterial agents and decrease side effects and drug resistance (Derendorf et al., 1997; Meibohm and Derendorf, 1997; MacGowan and Bowker, 2002; Toutain et al., 2002). In the veterinary field, the tissue-cage model is widely used for PK/PD studies and has been reported useful in several species, such as calves (Bengtsson and Greko, 2002), piglets (Zhang et al., 2014), goats (Sidhu et al., 2010), and rabbits (Xiong et al., 2016). However, there are few reported studies of macrolide drugs on PK/PD models. This may be because of disparity between *in vivo* plasma concentration and *in vitro* MICs. Macrolides have an extraordinary capacity to accumulate in leukocytes and lung tissues (Cao et al., 2006; Buret, 2010). The achieved plasma/serum concentrations of these medicines are lower than the *in vitro* MICs for major lung pathogens (Nowakowski et al., 2004). However, a recent report indicated that the dosage of tulathromycin can be documented using standard PK/PD concepts. The reason is that *in vitro* MIC value of tulathromycin against *P. multocida* and *M. haemolytica* in serum is much lower than in MHB. The present study provides support for standard PK/PD concepts that have sufficed to derive an appropriate dose with the crucial proviso that MIC have to be determined in serum for tulathromycin (Toutain et al., 2017).

*Pasteurella multocida*, a Gram-negative bacillus, is a commensal bacterium that resides in the upper respiratory tract of piglets and plays a role in piglet respiratory disease. It may cause a wide spectrum of diseases ranging from septicemia to pneumonia in cattle and swine (Dabo et al., 2007).

Pharmacokinetic studies of tulathromycin have been conducted in various species, including pigs (Wang et al., 2012; Villarino et al., 2013; Gajda et al., 2016). However, there have been no studies that applied PK and *ex vivo* PD principles to evaluate the antibacterial effects of tulathromycin in piglets.

The aim of this study was to estimate the PK/PD values required for bacteriostasis, bactericidal activity, and virtual bacterial eradication for tulathromycin in piglets after intramuscular (i.m.) and intravenous (i.v.) administration. These data may provide a basis for estimating optimal therapeutic dose regimens.

## Materials and Methods

### Antimicrobial Agent

Tulathromycin (Draxxin^®^ 100 mg/mL injectable solution; Zoetis, New York, NY, 10017, United States) was used for administration. Tulathromycin standard was purchased from Toronto Research Chemicals (Toronto, ON, Canada). Roxithromycin standard was provided by China Institute of Veterinary Drugs Control (Beijing, China).

### Bacterial Strain

Six *P. multocida* strains isolated from pigs and standard strain *P. multocida* CVCC430 were evaluated in this study. *P. multocida* strain CVCC430 was obtained from the China Veterinary Culture Collection Centre (Beijing, China). Six clinical strains (FS01-FS06) were provided by Foshan University. Strain CVCC430 and six strains of *P. multocida* clinical isolates were grown, sub-cultured, and quantified in Muller-Hinton II broth (Becton Dickinson, Sparks, MD, United States) and Tryptic Soy Agar (Guangdong Huankai) supplemented with defibrinated sheep blood (BTSA) at a 5% level.

### Tissue Cage Manufacture and Surgical Implantation

Tissue cage model was developed as previously described (Sidhu et al., 2003). Briefly, tissue cages were made from platinum-cured medical grade silicone tubing (SF Medical, Hudson, MA, United States) 6.5 cm in length with an outside and inside diameter 1.8 and 1.3 cm, respectively. Each cage had 24 inentical holes and the total exchange surface was 2.3 cm^2^. Under deep sedation and local infiltration anesthesia two tissue cages were implanted subcutaneously, one on each side of each animal’s neck. Into one of the two tissue cages 0.5 mL of 1% w/v sterile carrageenan solution in saline was injected. After a 4–5-weeks recovery phase, each tissue cage had become sealed with a thin layer of connective tissue; non-injected cages were filled with clear, yellowish fluid (transudate) and injected tissue cages with inflammatory fluid (exudate).

### Animals and Experimental Design

Twelve healthy piglets (Duroc × Landrace × Yorkshire; six male, six female) weighing 13–15 kg were used. Each animal had free access to antibiotic-free fodder and water.

The piglets were randomly divided into two groups (TB1 and TB2), with six piglets in each group. After tissue cages had been implanted, tulathromycin 2.5 mg/kg body weight was administered via the i.v. route to animals in groups TB1 and by i.m. route to animals in groups TB2. All animal studies were approved by the Committee on the Ethics of Animals of South China Agricultural University (Approval number 2015-04; March 11, 2015).

### Sample Collection

Serum samples were collected from a jugular vein before and at 0.083, 0.25, 0.5, 0.75, 1, 1.5, 2, 3, 4, 6, 8, 10, 12, 24, 48, 60, 72, 96, 120, 144, 168, 192, 216, 240, 264, 288, 312, 336, and 360 h after administration using 5-mL plastic tubes without anticoagulant. Serum samples were allowed to stand, protected from sunlight, at room temperature for 30 min, then placed on ice for 30 min. Serum samples were maintained under refrigeration pending centrifugation at 4000 × *g* at 4°C for 10 min and the samples were stored at -80°C. Transudate and exudate samples (1.0 mL) were collected into 2-mL disposable syringes then transferred immediately into 2-mL micro centrifuge tubes. Samples were collected before and at 1, 3, 6, 9, 12, 24, 36, 48, 72, 96, 120, 144, 168, 192, 216, 240, 264, 288, 312, 336, and 360 h after administration. All samples were maintained under refrigeration pending centrifugation at 4000 × *g* at 4°C for 10 min and were stored at -80°C. Sampling of serum, transudate, and exudate was carried out following a previously reported protocol (Aliabadi and Lees, 2002).

### Analysis Method

Tulathromycin concentrations in serum, transudate, and exudate were determined by a method adapted from Clothier et al. (2011). Briefly, 10 μL of roxithromycin internal standard solution (10 μg/mL) was added to 500-μL samples. Subsequently, to all samples, 490 μL acetonitrile was added and vortexed for 1 min and the samples were centrifuged at 12,000 × *g* for 10 min. After centrifugation, clear supernatant were filtered through a 0.22-μm nylon syringe filter (JinTeng Experiment Equipment Company). The sample was transferred into an injection vial for ultra-high-pressure liquid chromatography tandem mass spectrometry (UPLC-MS/MS) analysis with the injection volume set to 5 μL. The mobile phases consisted of A: 0.1% formic acid in water and B: acetonitrile at a flow rate of 0.3 mL/min. The mobile phase began at 10% B with a linear gradient to 50% B at 2 min, which was maintained for 2 min, followed by re-equilibration to 10% B. The limit of quantification (LOQ) was 1 ng/mL. Tulathromycin quantification was linear within the range 1–500 ng/mL and the linearity of the standard curve was *r*^2^ > 0.99. The recoveries of tulathromycin in serum, transudate, and exudate samples were >85%. Inter-assay and intra-assay variation as measured by %RSD were all <10%.

### Pharmacokinetics Analysis

Pharmacokinetic parameters were calculated for tulathromycin in serum, transudate, and exudate using WinNonlin 5.2.1 software (Pharsight Corporation, Mountain View, CA, United States). The concentration–time data from serum, transudate and exudate samples were submitted to non-compartmental analysis using the statistical moment approach. The linear trapezoidal rule was used to calculate the area under the concentration–time curve (AUC). Elimination half-life (*t*_1/2β_) was estimated by log–linear regression analysis. Pharmacokinetic parameters was expressed as arithmetic mean ± standard deviation (SD).

### *In Vitro* Susceptibility Studies

The minimum inhibitory concentration (MIC) and minimum bactericidal concentration (MBC) of tulathromycin against *P. multocida* were determined in serum and transudate by a broth microdilution assay according to Clinical and Laboratory Standards Institute [CLSI] (2013) reference methods. After determination of MIC and MBC, the serum was spiked with tulathromycin concentrations ranging from 0.25 to 64 MIC, before bacterial inoculation (5 × 10^5^ CFU/mL). The cultures were incubated at 35°C. The viable count was determined after incubation for 0, 2, 4, 6, 10, and 24 h. The limit of detection was 200 CFU/mL. Growth and sterility controls were also checked. All experimentations were performed in triplicate.

### *Ex Vivo* Antimicrobial Activity of Tulathromycin

The *ex vivo* antibacterial activity against *P. multocida* was determined in samples (serum, transudate, and exudate) obtained from piglets after administration of tulathromycin. An isolate of *P. multocida* CVCC430 was grown freshly from beads stored at -80°C on TSA. Five to eight colonies were used to inoculate 9 mL of MHB and the cultures were placed in shaking at 37°C overnight. *Ex vivo* bacterial time-kill curves were determined in serum, exudate, and transudate samples as described by Aliabadi and Lees (2002).

### Pharmacokinetic/Pharmacodynamic (PK/PD) Integration and Modeling

Using *in vitro* MIC data and *in vivo* PK parameters, the surrogate markers of antimicrobial activity, AUC_(0-24 h)_/MIC and *C*_max_/MIC, were determined for serum, exudate, and transudate after i.m. dosing of tulathromycin.

For PK/PD integration, the AUC_(0-24 h)_/MIC values were obtained on the basis of the area under the concentration-time curve over 24 h divided by the MIC, which was determined in biological fluid (serum, transudate, and exudate) for *P. multocida* CVCC430. The AUC_(0-24h)_ values were estimated by multiplying the average drug concentration at each time by the incubation period of 24 h. The relationship between the *ex vivo* AUC_(0-24h)_/MIC ratio and the log_10_ difference between the initial bacterial count (in number of CFU/mL) and the bacterial count after 24 h of incubation was established for serum, exudate and transudate by using the sigmoid inhibitory *E*_max_ model. This model was described by the following equation:

$$E=E_{\max}-(E_{\max}-E_{0})\times \frac{C_{e}^{N}}{EC_{50}^{N}+C_{e}^{N}}$$

where *E* is the antibacterial effect measured as the change in log_10_ CFU/mL in serum, exudate, or transudate after 24-h incubation compared with the initial log_10_ CFU/mL; *E*_max_ is the corresponding bacterial growth in the absence of drug (control samples); *E*_0_ is the maximum antibacterial growth inhibition determined as difference in log_10_ CFU/mL in samples over 24-h incubation; EC_50_ is the AUC_(0-24 h)_/MIC value producing 50% of the maximal antibacterial effect; *C*_e_ is the AUC_(0-24 h)_/MIC in the effect compartment (*ex vivo* site), and N is the Hill coefficient. These PD parameters were calculated using the non-linear WinNonlin regression program (Pharsight Corporation).

Three levels of antibacterial effect of tulathromycin were quantified from the sigmoid *E*_max_ equation by determining AUC_(0-24 h)_/MIC values required for bacteriostatic effect (*E* = 0, no change form initial inoculum count after 24-h incubation), bactericidal effect (*E* = -3, 99.9% reduction of the original inoculum count after 24-h incubation), and virtual bacterial eradication (*E* = -4, 99.99% reduction in count) in each of the analyte fluids (serum, exudate, and transudate).

### Computation of Dose

For an antimicrobial drug, the dose required for a given antibacterial activity is provided by the equation (Toutain et al., 2007):

$$Dose_{\left(\mathrm{for}\;5\;\;\mathrm{days}\right)}=\frac{{\mathrm{Cl}}_{\left(\mathrm{for}\;5\;\;\mathrm{days}\right)}\times \mathrm{factor}\times {\mathrm{MIC}}_{90}(\mathrm{or}{\mathrm{MIC}}_{50})}{\mathrm{fu}\times F}$$

where Dose _(for5 days)_ is a dose to guarantee 5 days of antimicrobial efficacy; Cl_(for5 days)_ is the serum clearance for 5 days of treatment; factor is the dimensionless numerical value of AUC/MIC; MIC_90_ is the 90th percentile of the MIC distribution; MIC_50_ is the 50th percentile of the MIC distribution (Sweeney et al., 2017); *F* is the absolute bioavailability; fu is the free drug fraction, ranging from 0.53 to 0.68 (Anonymous, 2005).

### Statistical Analyses

Statistical analyses were undertaken by analysis of variance, and significant differences, when they occurred, were examined using Bonferroni’s correction for intergroup comparisons. Differences were accepted as significant for *P*-values < 0.05.

## Results

### Pharmacokinetics of Tulathromycin

**FIGURE 1:**
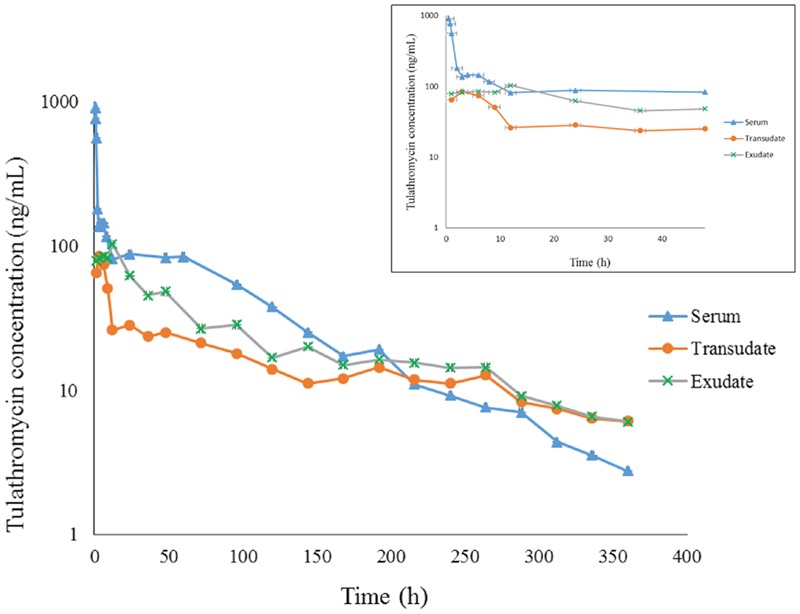
The concentration-time curve for tulathromycin in serum, transudate and exudate after i.v. administration at a dosage of 2.5 mg/kg body weight (for clarity the small graph depicts the drug concentrations in the first 48 h post-administration).

**FIGURE 2:**
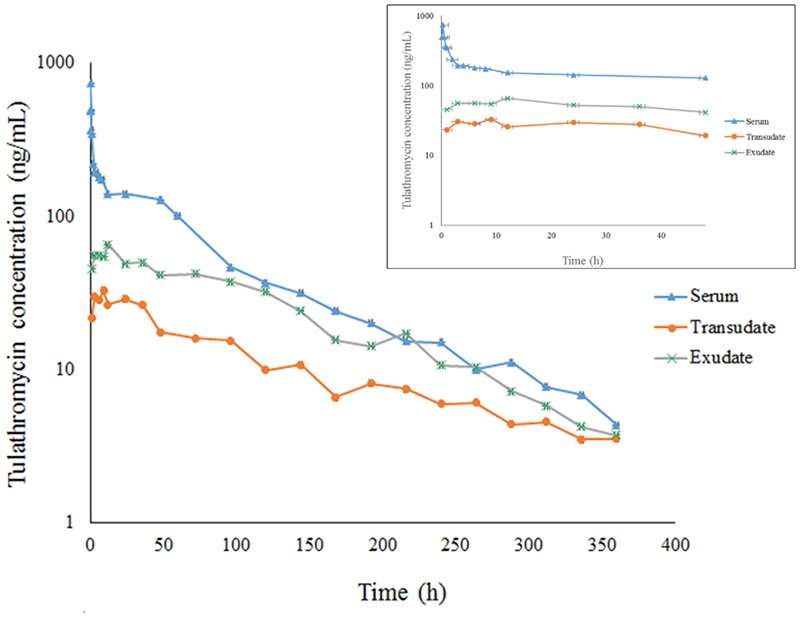
The concentration-time curve for tulathromycin in serum, transudate and exudate after i.m. administration at a dosage of 2.5 mg/kg body weight (for clarity the small graph depicts the drug concentrations in the first 48 h post-administration).

Serum concentration vs. time data after i.v. and i.m. administration were analyzed by non-compartmental model. The serum concentration–time profiles are illustrated in **Figures 1, 2**. Pharmacokinetics parameters are presented in **Tables 1–3**. Absorption was rapid after i.m. administration and reached maximum serum concentrations 0.74 ± 0.22 μg/mL by 0.25 h. The drug was slowly eliminated with a half-life 63.55 ± 8.20 h.

**Table 1:** The pharmacokinetic parameters (non-compartmental analysis) of tulathromycin in serum after i.v. or i.m. administration at a dosage of 2.5 mg/kg (mean ± SD, *n* = 6).

	Intravenous		Intramuscular	
Variable (units)	Mean	*SD*	Mean	*SD*
*C*_max_ (μg/mL)	-	-	0.74	0.22
*T*_max_ (h)	-	-	0.25	0.00
*t*_1/2β_ (h)	71.33	22.92	63.55	8.20
AUC_(0-last)_ (μg⋅h/mL)	15.95	4.56	16.32	2.40
AUC_(0-∞)_ (μg⋅h/mL)	16.31	4.40	16.72	2.48
Cl (L/kg/h)	0.16	0.04	-	-
Cl/F (L/kg/h)	-	-	0.15	0.02
MRT_(0-last)_ (h)	72.52	11.40	80.74	3.74
*F* (%)	-	-	104.27	12.84

*C_*max*_, maximum concentration in serum; *T*_*max*_, time to achieve maximum concentration; *t*_*1/2*β_, elimination half-life; AUC_*(0*-*last)*_, area under serum concentration–time curve to last sampling time; AUC_*(0*-∞*)*_, area under serum concentration–time curve to infinity; MRT_*(0*-*last)*_, mean residence time to last sampling time; Cl, clearance; Cl/F, clearance scaled by bioavailability; *F*, absolute bioavailability.*

**Table 2:** The pharmacokinetic parameters (non-compartmental analysis) of tulathromycin in exudate and transudate after i.v. administration at a dosage of 2.5 mg/kg (mean ± SD, *n* = 6).

	Transudate		Exudate	
Variable (units)	Mean	*SD*	Mean	*SD*
*C*_max_ (μg/mL)	0.10	0.03	0.14	0.06
*T*_max_ (h)	4.50	1.64	8.50	3.51
*t*_1/2β_ (h)	69.99	13.36	72.78	25.09
AUC_(0-last)_ (μg⋅h/mL)	5.98	1.89	9.11	3.16
AUC_(0-∞)_ (μg⋅h/mL)	8.47	3.03	8.46	3.01
MRT_(0-last)_ (h)	128.95	20.16	110.21	11.10

*C_*max*_, maximum concentration in serum; *T*_*max*_, time to achieve maximum concentration; *t*_*1/2*β_, elimination half-life; AUC_*(0*-*last)*_, area under serum concentration–time curve to last sampling time; AUC_*(0*-∞*)*_, area under serum concentration–time curve to infinity; MRT_*(0*-*last)*_, mean residence time to last sampling time.*

**Table 3:** The pharmacokinetic parameters (non-compartmental analysis) of tulathromycin in exudate and transudate after i.m. administration at a dosage of 2.5 mg/kg (mean ± SD, *n* = 6).

	Transudate		Exudate	
Variable (units)	Mean	*SD*	Mean	*SD*
*C*_max_ (μg/mL)	0.05	0.01	0.11	0.03
*T*_max_ (h)	21.50	9.75	8.00	3.63
*t*_1/2β_ (h)	104.94	29.24	63.96	18.97
AUC_(0-last)_ (μg⋅h/mL)	3.89	1.06	8.32	3.33
AUC_(0-∞)_ (μg⋅h/mL)	4.42	1.17	8.66	3.38
MRT_(0-last)_ (h)	113.80	15.89	104.99	14.45

*C_*max*_, maximum concentration in serum; *T*_*max*_, time to achieve maximum concentration; *t*_*1/2*β_, elimination half-life; AUC_*(0*-*last)*_, area under serum concentration–time curve to last sampling time; AUC_*(0*-∞*)*_, area under serum concentration–time curve to infinity; MRT_*(0*-*last)*_, mean residence time to last sampling time.*

Transudate and exudate drug concentration–time data were analyzed by non-compartmental model. The non-compartmental pharmacokinetic parameters are presented in **Tables 2, 3**. Tulathromycin penetration into transudate and exudate was similar for both fluids. The time vs. mean transudate and exudate concentration data are shown in **Figures 1, 2**. After i.m. administration of tulathromycin, transudate and exudate drug concentrations were lower than serum levels. *C*_max_ was slightly but not significantly higher in exudate compared to transudate. Moreover, *C*_max_ was reached at 21.5 h for transudate and 8 h for exudate.

Mean tulathromycin AUC_(0∼last)_ values for transudate (3.89 μg⋅h/mL) and exudate (8.32 μg⋅h/mL) were significantly lower than that for serum (16.32 μg⋅h/mL) after i.m. administration (both *P* < 0.05). However, the rate of elimination of tulathromycin from transudate was slower than from serum; mean half-life in transudate and exudate was 104.94 and 63.96 h, respectively.

Longer persistence of drug in exudate and transudate compared to serum was further indicated by MRT values for transudate (113.80 h) and exudate (104.99 h). These were significantly higher than values obtained in serum (80.74 h) after i.m. administration of tulathromycin.

### MIC and MBC Values for Tulathromycin

**FIGURE 3:**
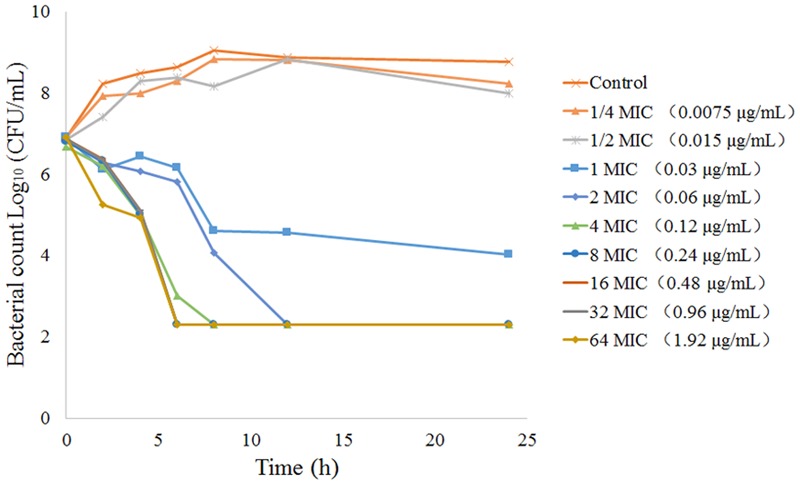
*In vitro* time-kill curves for tulathromycin concentration range 0.25-64 MIC against *P. multocida* CVCC430 in serum.

The MICs and MBCs of tulathromycin against *P. multocida* in MHB and serum are shown in **Table 4**. The average MIC and MBC in serum were 0.04 and 0.10 μg/mL, respectively. The MICs of tulathromycin against *P. multocida* CVCC430 in transudate and exudate were all 0.03 μg/mL. The MBCs in transudate and exudate were all 0.06 μg/mL. *In vitro* killing curves for tulathromycin against *P. multocida* CVCC430 were established in serum for multiples of MIC (0.25–64 MIC). Bacterial count determined at different times after inoculation is shown in **Figure 3**. Tulathromycin concentration of 0.25 and 0.5 MIC failed to inhibit bacterial growth whereas 1 MIC resulted in a slight reduction of bacterial counts. Increasing the drug concentration to 2 MIC showed bactericidal effect whereas increasing to 8 MIC achieved sterilization in serum.

**Table 4:** The MICs of tulathromycin against *P. multocida* in MHB and serum.

*P. multocida* strain	MHB		Serum		MHB : Serum MIC ratio
	MIC (μg/mL)	MBC (μg/mL)	MIC (μg/mL)	MBC (μg/mL)	
CVCC430	0.25	0.25	0.03	0.06	8.33
FS-01	0.5	1	0.03	0.06	16.67
FS-02	0.5	1	0.06	0.25	8.33
FS-03	0.5	1	0.03	0.06	16.67
FS-04	0.5	1	0.03	0.06	16.67
FS-05	0.5	2	0.03	0.06	16.67
FS-06	2	4	0.06	0.125	33.33

### PK/PD Integration for Tulathromycin

The PK/PD integration parameters that established the surrogates AUC_(0-last)_/MIC, AUC_(0-∞)/_MIC, and *C*_max_/MIC are presented in **Table 5**. The serum AUC_(0-last)_/MIC and C_max_/MIC values after i.m. administration of tulathromycin were 407.91 h and 18.50, respectively. The AUC_(0-last)_/MIC ratios after i.m administration were 129.55 h in transudate and 277.27 h in exudate. These parameters indicated that serum concentrations of tulathromycin would be predicted to have antibacterial activity against *P. multocida*.

**Table 5:** *In vivo* PK/PD integration parameters for tulathromycin after i.v. or i.m. administration and *in vitro* measurement of MIC (mean ± SD, *n* = 6).

PK/PD integration	Serum		Transudate		Exudate	
	Mean	*SD*	Mean	*SD*	Mean	*SD*
Intravenous						
AUC_(0-last)_/MIC (h)	398.67	114.01	282.25	100.92	281.86	100.32
AUC_(0-last)_/MBC (h)	159.47	45.60	141.13	50.46	140.93	50.16
*C*_max_/MIC	-	-	3.38	1.11	4.82	2.05
*C*_max_/MBC	-	-	1.69	0.56	2.41	1.02
AUC_(0-∞)_/MIC (h)	407.80	110.02	199.40	63.05	303.68	105.27
AUC_(0-∞)_/MBC (h)	163.12	44.01	99.70	31.v52	151.84	52.63
Intramuscular						
AUC_(0-last)_/MIC (h)	407.91	60.12	129.55	35.48	277.27	111.08
AUC_(0-last)_/MBC (h)	163.17	24.05	64.78	17.74	138.64	55.54
*C*_max_/MIC	18.50	5.53	1.76	0.48	3.82	1.04
*C*_max_/MBC	7.40	2.21	0.88	0.24	1.91	0.52
AUC_(0-∞)_/MIC (h)	417.90	62.09	147.29	38.93	288.66	112.72
AUC_(0-∞)_/MBC (h)	167.16	24.83	73.65	19.47	144.33	56.36

*MIC (serum) = 0.04 μg/mL; MBC (serum) = 0.10 μg/mL; MIC (transudate, exudate) = 0.03 μg/mL; MBC (transudate, exudate) = 0.06 μg/mL.*

### Correlation of PK/PD Parameters with Effectiveness

**FIGURE 4:**
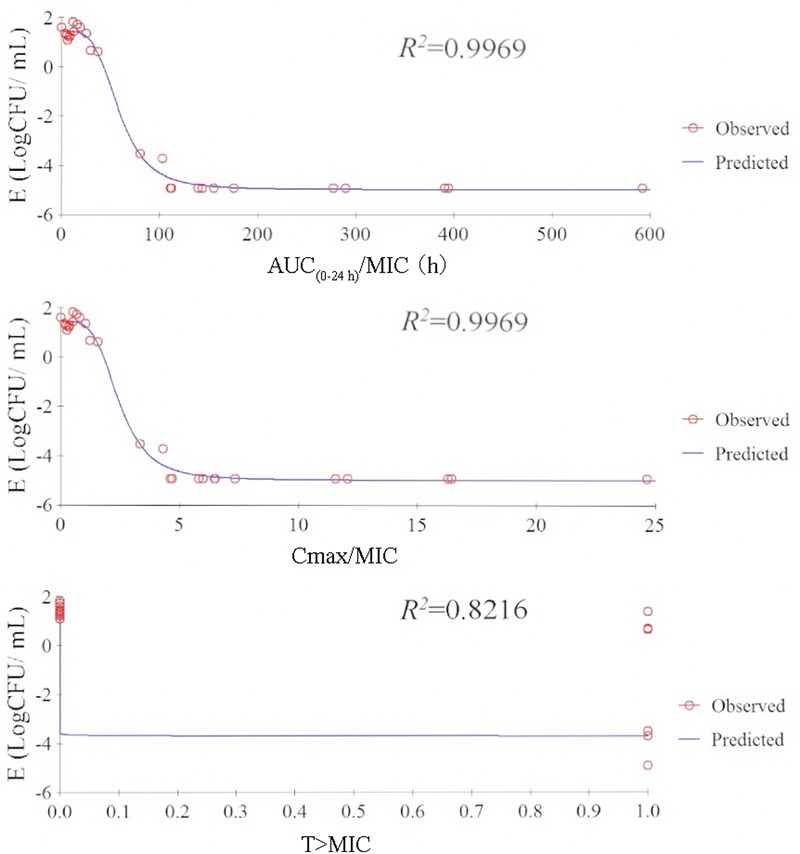
Relationship between PK/PD parameters based MIC for *P. multocida* and Log10 CFU/mL after 24-h incubation compared with the initial Log_10_ CFU/mL by using sigmoid Emax model in serum.

The relationship between PK/PD index [AUC_(0-24h)_/MIC, *C*_max_/MIC, %T > MIC] and the antibacterial effectiveness (Δlog_10_ CFU/mL: the change in log_10_ CFU/mL in serum after 24-h incubation compared with the initial log_10_ CFU/mL) are illustrated in **Figure 4**. The pharmacokinetic-pharmacodynamic (PK/PD) index [AUC_(0-24 h)_/MIC and *C*_max_/MIC] were best described the effectiveness of tulathromycin against *P. multocida* (*R*^2^ = 0.9969) by using sigmoid *E*_max_ model.

### *Ex Vivo* Antibacterial Activity of Tulathromycin

**FIGURE 5:**
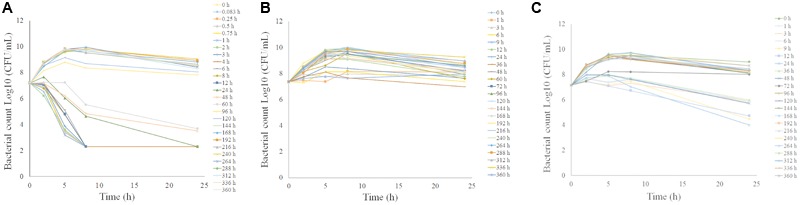
Ex vivo antibacterial activity of tulathromycin against P.multocida in serum **(A)**, transudate **(B)**, exudate **(C)** after i.m. administration at a dosage of 2.5 mg/kg.

The *ex vivo* antibacterial time-kill curves for tulathromycin against *P. multocida* CVCC430 in serum, transudate, and exudate are illustrated in **Figure 5**. In serum samples collected between 0.083 and 60 h after administration, tulathromycin showed effective bactericidal action (3-Log10 CFU/mL or 4-Log10 CFU/mL reduction) after 8-h incubation (**Figure 5A**). No inhibition of bacterial growth was observed after 96 h.

The *ex vivo* antibacterial activity was different for transudate and exudate. For samples collected from transudate, tulathromycin exerted slight inhibition of bacterial growth collected at 36 h and no inhibition of bacterial growth was obtained for transudate collected at other time-points (**Figure 5B**). In exudate samples collected at 24 h, tulathromycin exerted effective bactericidal action after 24-h incubation (**Figure 5C**). Exudate samples exerted slight inhibition of bacterial growth collected at 1, 3, 6, 9, 12, 36, and 48 h. No bacteriostatic were obtained for exudate collected at other time-points.

### *Ex Vivo* PK/PD Integration and Modeling

The PK/PD integration of *ex vivo* tulathromycin data for serum, transudate, and exudate are presented in **Table 6**. The calculated mean AUC_(0-24 h)_/MICs for serum that produced bacteriostatic, bactericidal effect, and virtual bacterial eradication were 44.55, 73.19, and 92.44 h, respectively. For transudate and exudate, slightly lower values were obtained.

**Table 6:** Integration of pharmacokinetic and pharmacodynamics data obtained for tulathromycin after administration of 2.5 mg/kg in piglets (*n* = 6).

Parameter	Serum	Transudate	Exudate
*E*_max_(log_10_ CFU/mL)	1.60	1.08	1.85
EC_50_ (h)	58.80	13.70	33.55
*E*_0_ (log_10_ CFU/mL)	-4.91	-0.40	-3.16
Slope (N)	4.03	1.88	15.55
AUC_(0-24 h)_/MIC for bacteriostatic action (h)	44.55	23.17	32.42
AUC_(0-24 h)_/MIC for bactericidal action (h)	73.19		41.85
AUC_(0-24 h)_/MIC for virtual bacterial eradication (h)	92.44		

*E_*max*_ is the corresponding bacterial growth in the absence of drug (control samples); *E*_*0*_ is the maximum antibacterial growth inhibition determined as difference in log_*10*_ CFU/mL in samples over 24-h incubation; EC_*50*_ is the AUC_*(0*-*24 h)*_/MIC value producing 50% of the maximal antibacterial effect; N is the Hill coefficient.*

The MIC_50_ or MIC_90_ against *P. multocida* in artificial broth has been reported by Sweeney et al. (2017). The MHB MIC was then transformed into MIC in serum by a factor of 16.67. Based on the AUC_(0-24 h)_/MIC values for tulathromycin and the MIC in serum, the calculated corresponding doses of tulathromycin against *P. multocida* are presented in **Table 7**.

**Table 7:** The dosage of tulathromycin for achieving corresponding antibacterial effect following i.m. administration.

	Antimicrobial efficacy of dosage (mg/kg b.w.)	MIC_50_ in serum		MIC_90_ in serum	
		0.06 μg/mL	0.12 μg/mL	0.12 μg/mL	0.24 μg/mL
Serum	Bacteriostatic action	3.19	6.39	6.39	12.78
	Bactericidal action	5.25	10.49	10.49	20.99
	Virtual bacterial eradication	6.63	13.25	13.25	26.51
Transudate	Bacteriostatic action	1.66	3.32	3.32	6.64
	Bactericidal action				
	Virtual bacterial eradication				
Exudate	Bacteriostatic action	2.32	4.65	4.65	9.30
	Bactericidal action	3.00	6.00	6.00	12.00
	Virtual bacterial eradication				

*Dosage was computed by considering breakpoint values of the PK/PD index as obtained in serum, transudate or exudate; MIC_*90*_ and MIC_*50*_ as obtained from conventional MHB were transformed into equivalent MIC in serum by dividing reported values by a scaling factor of 16.67 as determined in the present experiment.*

## Discussion

The pharmacokinetics of tulathromycin has been studied in cattle, piglets, goats, bison, and deer following i.m. and i.v. administration at label dose. In the present study in piglets following i.v. and i.m. administration mean half-life (*t*_1/2β_) was 71.33 and 63.55 h and AUC_(0-last)_ 15. 95 and 16.32 μg⋅h/mL. These results are similar to those reported by Benchaoui et al. (2004). Following i.m. administration to piglets at 2.5 mg/kg body weight, *C*_max_ (0.74 μg/mL) and *T*_max_ (0.25 h) suggest that tulathromycin was characterized by rapid absorption and eliminated slowly. The AUC_(0-24 h)_ and *C*_max_ values of transudate and exudate were much lower than in serum. In addition, transudate and exudate tulathromycin concentrations were always lower than those in serum. The main reason for this is that tulathromycin accumulates to a high extent in neutrophils and macrophages in blood (Siegel et al., 2004), whereas transudate and exudate do not contain or contain only a small number of cells, which may reduce drug concentrations in both these fluids.

The MIC of tulathromycin against *P. multocida* was 0.06–4 μg/mL in MHB (Godinho et al., 2005). There are limited data available on the MIC in serum and transudate. In a recent study, Toutain et al. (2017) suggested that MICs should be determined in biological fluid such as serum when establishing PK/PD relationships. In the present study, the MIC and MBC values for tulathromycin were determined not only in MHB but also in serum and transudate, which were determined according to CLSI. The average MIC and MBC of tulathromycin against *P. multocida* in serum were 0.04 and 0.10 μg/mL, respectively. Similar values for tulathromycin were also reported in cattle (Toutain et al., 2017). However, in this study, the average MIC for *P. multocida* was 0.68 μg/mL in MHB. This disparity may be because of different culture conditions. Studies have reported that the changes of local conditions would lead to changes in MIC (Reese et al., 2004). Similar observations have been reported for azithromycin, erythromycin, and clarithromycin etc., which is due to change in the culture environment (Ednie et al., 1998).

Choosing an appropriate clinical dose of antimicrobial agent is crucial for optimizing clinical efficacy and the prevention of bacterial drug resistance (Toutain et al., 2002). PK/PD approaches have been successfully applied in selection of the dose regimens of antibiotics. For tulathromycin, the intracellular concentrations are higher than plasma concentrations (Cox et al., 2010). Prior research has shown that tulathromycin exerts concentration-dependent bactericidal activity against *Haemophilus somnus in vitro* (Reese et al., 2004). In addition, a recent study has demonstrated that the use of AUC/MIC as a PK/PD predictor of efficacy of tulathromycin is feasible for *M. haemolytica* and *P. multocida* (Toutain et al., 2017). In the present study the AUC/MIC data were regarded as a promising predictor for the efficacy of tulathromycin by using the sigmoid *E*_max_ equation with the reduction in bacterial numbers after 24-h incubation. For bacteriostatic, bactericidal effect, and virtual bacterial eradication *ex vivo*, serum AUC_(0-24 h)_/MIC values for tulathromycin were 44.55, 73.19, and 92.44 h, respectively. Compared to serum AUC_(0-last)_/MIC values, slightly lower values were obtained in transudate and exudate. However, the present findings only relate to a single strain of *P. multocida*. Further to develop an effective dose determination against respiratory infections caused by *P. multocida*, additional data are required. Data on MIC_90_ for a reasonable number of strains of this species are needed.

Different dosage regimens should depend on the type of use of an antibiotic (Ferran et al., 2011). In this study, a dosage of 13.25 mg/kg was recommended to achieve a total eradication of *P. multocida* for a MIC_90_. This result showed rather higher than the recommended dose (2.5 mg/kg). That might be due to the computed dose was computed with a MIC_90_ corresponding to a worst case scenario and the dose would be lower to base upon the *P. multocida* MIC distribution using a Monte Carlo simulation (MCS). However, in the framework of the prudent use of antimicrobial, the dose for a bacteriostatic effect that could be enough in most instances for long-acting antimicrobial metaphylaxis, which associated with a significant reduction in disease incidence (Stanton et al., 2013; Teixeira et al., 2017; Toutain et al., 2017). Hence, we also computed the doses for the bacteriostatic effect of *P. multocida* were 6.39, 3.32, and 4.65 mg/kg using serum, transudate and exsudate PK/PD breakpoints, respectively.

One limitation of *ex vivo* models is that the PK parameters do not take into account the differences between *in vitro* and *in vivo* conditions. *In vivo* the pathogens was exposure to a gradient of drug concentration instead of a fixed concentration of drug in a defined time in *ex vivo* model. Moreover, *ex vivo* models do not consider the effect of body clearance and the role of the immune system of the host (Aliabadi and Lees, 2002). Because of possible *in vivo* and *ex vivo* differences in antimicrobial activity, the dose obtained by the present method might not be recommended for clinical use but might be suitable for evaluation in clinical trials.

The second possible limitation of our results is that there was no population data for the PK part of the dosage regimen computation. In this study, the recommended doses of tulathromycin were computed using mean values of PK parameters and MIC_50_ or MIC_90_. However, the breeds of pigs, age, physical status etc. could affect the prediction of dose effectiveness and toxicity. Hence, the pharmacokinetics of the drug in the clinical population should be conducted in the future. It would be preferable to calculating the dose based upon the MIC distribution for the bacterial species and the MCS should be applied to take these factors into consideration.

The PK/PD integration method described in this study may provide the basis for optimizing clinical dosing regimens. In this study, we concluded that the dose of tulathromycin for a bacteriostatic effect and eradication of *P. multocida* as computed using the value of the PK/PD breakpoint obtained in serum were 6.39 and 13.25 mg/kg, respectively. Of course, the results of this model need to be combined with the population pharmacokinetic data and be validated by either naturally diseased animals or disease models. However, it is of great significance to further understand the analysis of tulathromycin PK/PD relationships.

## Author Contributions

BF and QZ: conceived and designed the experiments. QW, WL, and YH: performed the experiments. PY: analysis data. QZ: drafted the manuscript. HD, YL, and GZ: contributed to the revision. All authors read and approved the final manuscript.

## Conflict of Interest Statement

The authors declare that the research was conducted in the absence of any commercial or financial relationships that could be construed as a potential conflict of interest.
